# Neonatal Hypoglycemia and Brain Vulnerability

**DOI:** 10.3389/fendo.2021.634305

**Published:** 2021-03-16

**Authors:** Laura Costanza De Angelis, Giorgia Brigati, Giulia Polleri, Mariya Malova, Alessandro Parodi, Diego Minghetti, Andrea Rossi, Paolo Massirio, Cristina Traggiai, Mohamad Maghnie, Luca Antonio Ramenghi

**Affiliations:** ^1^ Neonatal Intensive Care Unit, Department Mother and Child, IRCCS Istituto Giannina Gaslini, Genoa, Italy; ^2^ Department of Neurosciences, Rehabilitation, Ophthalmology, Genetics, Maternal and Child Health (DINOGMI), University of Genoa, Genoa, Italy; ^3^ Department of Health Sciences (DISSAL), University of Genoa, Genoa, Italy; ^4^ Neuroradiology Unit, Istituti di Ricovero e Cura a Carattere Scientifico (IRCCS) Istituto Giannina Gaslini, Genoa, Italy; ^5^ Department of Pediatrics, IRCCS Istituto Giannina Gaslini, Genoa, Italy

**Keywords:** neonatal hypoglycemia, brain damage, glucose homeostasis, glucose sensing neurons, brain energetics

## Abstract

Neonatal hypoglycemia is a common condition. A transient reduction in blood glucose values is part of a transitional metabolic adaptation following birth, which resolves within the first 48 to 72 h of life. In addition, several factors may interfere with glucose homeostasis, especially in case of limited metabolic stores or increased energy expenditure. Although the effect of mild transient asymptomatic hypoglycemia on brain development remains unclear, a correlation between severe and prolonged hypoglycemia and cerebral damage has been proven. A selective vulnerability of some brain regions to hypoglycemia including the second and the third superficial layers of the cerebral cortex, the dentate gyrus, the subiculum, the CA1 regions in the hippocampus, and the caudate-putamen nuclei has been observed. Several mechanisms contribute to neuronal damage during hypoglycemia. Neuronal depolarization induced by hypoglycemia leads to an elevated release of glutamate and aspartate, thus promoting excitotoxicity, and to an increased release of zinc to the extracellular space, causing the extensive activation of poly ADP-ribose polymerase-1 which promotes neuronal death. In this review we discuss the cerebral glucose homeostasis, the mechanisms of brain injury following neonatal hypoglycemia and the possible treatment strategies to reduce its occurrence.

## Introduction

Hypoglycemia is a common condition in the neonatal population (1). It has a significant prevalence in at risk infants, with that of 47% in large-for-gestational age (LGA) infants, 52% in small-for-gestational age (SGA) infants, 48% in neonates of diabetic mothers, and 54% in late preterm infants (2). In infants born before 33 weeks, the prevalence of hypoglycemia is nearly 34% (3).

A transient reduction in blood glucose values immediately after birth is part of a transitional metabolic adaptation that generally resolves within the first hours of life as glucose levels gradually increase to reach adult values (blood glucose > 70 mg/dl) within the first 72 to 96 h (4, 5). However, a minority of neonates experience a prolonged and severe hypoglycemia, usually associated with specific risk factors (6).

Although adverse effects on the neurodevelopmental outcome have been proven for severe and persistently low glucose levels (7–10), the long-term significance of transient asymptomatic neonatal hypoglycemia remains unclear. Additionally, while a “neurologically safe” blood glucose numerical threshold has not yet been identified, guidelines for the optimization of detection and prompt treatment of hypoglycemic neonates have been empirically established to avoid neurologic sequelae (11). A summary of the main expert opinions on the definition of neonatal hypoglycemia is presented in **Table 1**. In spite of the inhomogeneous definitions, no evidence of the superiority of hypoglycemia management according to different blood glucose thresholds has currently been established.

**Table 1:** Definition of neonatal hypoglycemia in the first 72 h from birth, according to the more widespread recommendations.

Definition of neonatal hypoglycemia (mg/dl)						
Institution, year	Time from birth					
	0–2 h	2–4 h	4–24 h	24–48 h	48–72 h	>72 h
AAP, 2011 (12)	<40 mg/dl		<45 mg/dl		<60 mg/dl	
ABM, 2014 (13)	<28 mg/dl	<40 mg/dl			<48 mg/dl	
PES, 2015 (14)	<50 mg/dl				<60 mg/dl	
BAPM, 2017 (15)	<45 mg/dl if symptomatic <36 mg/dl in asymptomatic at risk infants					
CPS 2019 (16)	<47 mg/dl					
SNG, 2019 (17)	<47 mg/dl					<54 mg/dl

AAP, American Academy of Pediatrics; BAPM, British Association of Perinatal Medicine; ABM, Academy of Breastfeeding Medicine; CPS, Canadian Pediatric Society; SNG, Swedish national guidelines; PES, Pediatric Endocrine Society.

The aim of this review is to discuss the cerebral glucose homeostasis, the mechanisms of brain injury following neonatal hypoglycemia and the possible treatment strategies to reduce its occurrence.

## Transition of Glucose Homeostasis

Glucose is the main energy source for fetal development. During pregnancy, its homeostasis entirely depends on continuous glucose supply from the maternal circulation (18). Glucose transportation across the placenta is mediated by facilitate diffusion, dependent on the maternal-fetal concentration gradient (19, 20). About the 70% of the maternal glucose is allocated to the fetus while the 30% is consumed by the placenta (21). Fetal glucose metabolism is regulated by fetal insulin production, which increases with pregnancy progression and enhances glucose utilization by insulin-sensitive tissues, including skeletal muscle, liver, heart, and adipose tissue (22).

At birth, the interruption of glucose transfer causes a prompt feedback of neonatal glucose control leading to a consistent increase in plasma catecholamines, glucagon (and glucagon receptors) and cortisol, and a decrease in plasma insulin levels (23). These endocrine changes are essential for inducing hepatic glycogenolysis and gluconeogenesis thus maintaining glucose homeostasis. In addition, lipolysis, fatty acid oxidation, and proteolysis actively concur to sustain blood glucose levels in the neonatal period (24). This metabolic pattern allows the prompt activation of catabolic processes and the utilization of endogenous energy stores (25).

Hepatic glycogenolysis is the fastest mechanism that allows an increase of blood glucose levels after birth. The high rate of glycogenolysis leads to hastened depletion of hepatic glycogen stores, especially in preterm infants in which liver glycogen deposits are limited. Gluconeogenesis is not immediately effective after birth due to the initial low enzymatic activity of this metabolic pathway. Gluconeogenesis slowly starts after some hours from birth and reaches its maturation after 12 h (26).

Glucose oxidation supports about 70% of the energy requirement of the brain. Ketone bodies and lactate are important alternative fuels to reduce the glucose requirement. Hepatic ketogenesis markedly increases during the first hours from birth, to provide alternative fuels for brain metabolism in term infants. However this metabolic pattern is severely limited in preterm infants due to a lack of fat stores in adipose tissue, which eventually results in failure of lipolysis (27).

During the first days of life interferences of neonatal glucose homeostasis may happen, especially in case of limited metabolic stores or increased energy expenditure (28). Most studies have shown that premature infants are more vulnerable than full-term infants to remain normoglycemic in the first week of life. This depends on several mechanism such as insulin resistance, deficient proinsulin processing by pancreatic beta cells (29), abnormal glucagon response (30), and lack of suppression of hepatic glucose release following intravenous glucose infusion that have been suggested to contribute to hyperglycemia. Predisposition to hypoglycemia is due to low glucose-6-phosphatase activity (31), the existence of an incompletely coordinated counter-regulatory system (32), increased basal metabolism of glucose, a lower capacity for production of alternative energy sources from their already insufficient stores (27), and presence of clinical conditions associated with hypoglycemia, such as perinatal asphyxia, hypoxia, sepsis, and hypothermia. As a consequence, it is essential to provide exogenous glucose at birth, especially in preterm infants (33).

## Brain Energetics and Glucose Sensing Neurons

The largest proportion of brain energy is consumed for neuronal computation, information processing, and maintaining ion gradients across neuronal membranes. Glucose metabolism provides several precursors as well as the energy for the biosynthesis of neurotransmitters (34). Brain function impairments caused by hypoglycemia seems to begin prior to any detectable drop in overall brain ATP. Neuronal activity may be suppressed as an energy conserving strategy. The release of ADP, induced by hypoglycemia, suppresses excitatory postsynaptic potential, and ATP produced by glycolysis (during euglycemia) has a special role in maintaining glutamatergic neurotransmission (35).

Production of glutamate vesicles is a glucose-dependent process. The membrane of glutamate vesicles contains enzymes responsible for several ATP generating steps of glycolysis, so the rate of glutamate refilling these vesicles is reduced in the absence of glucose resulting in the inhibition of all neuronal glutamate activity. With severe hypoglycemia, ATP levels decline simultaneous to glycogen store depletion.

Glucose homeostasis is based on an accurate and prompt detection of blood glucose variations. Glucose sensing neurons are a specialized group of neurons which modify their electrical activity according to extracellular glucose levels (36). Among them, glucose-excited neurons increase their activity in response to an increase of interstitial glucose levels while glucose-inhibited neurons reduce their firing rate. As glucose concentrations decrease, GI neurons are excited, whereas GE neurons decrease their activity (37). Glucose sensing neurons are mainly situated in the hypothalamic nuclei, the prefrontal cortex, the hippocampus, the paraventricular thalamus, and the amygdala (38–40).

The glucose level in the brain is about 30% of the blood level, as the presence of the blood-brain-barrier (BBB) prevents its free diffusion. The glucose supply to the brain is ensured by the high affinity of the glucose transporter type 1 (GLUT1) of brain endothelial cells (41). Glucose subsequently enter the neurons through GLUT1 and the glucose transporter type 3 (GLUT3) (42).

Some studies demonstrated that hypothalamic glucose levels do not fluctuate significantly following meals. Conversely, glucose levels in the arcuate nucleus were observed to slightly increase after fasting due to an increased permeability of the BBB. Thus, only profound alterations of blood glucose levels induce changes in brain glucose levels (43). Nevertheless, the presence of specialized neurons able to detect changes above 2.5 mM suggests that, at least in confined areas (such as those around fenestrated capillaries in the BBB), glucose levels may be increased to levels closer to that of the blood. The modification in cerebral fluid glucose caused by a change in blood sugar was observed to be delayed and dampened. BBB permeability to blood glucose changes depend on various pathophysiological conditions, including hyperglycemia and uncontrolled diabetes mellitus (44).

As the maintenance of a constant and adequate supply of sugar for the brain is a priority, it is obvious that the ability to detect changes in blood glucose levels by sensitive neurons situated behind the BBB is not useful to glucose homeostasis because it only foresees changes in brain glucose. It is likely that this ability serves more as a “fail safe system”. These neurons can detect basic conditions to compare information and create anticipatory responses (43).

Besides neurons, astrocytes have also been reported to be involved in glucose sensing, and due to their role in blood flow regulation, they may regulate it according to the neuronal metabolic state (45).

## Hypoglycemic Brain Damage

Many factors have been implicated in neuronal death induced by severe hypoglycemia. This event is not only a consequence of energy failure but also as a result of a sequence of events induced by hypoglycemia. Interestingly, correction of plasma glucose concentration alone does not interrupt this process. The main events induced by hypoglycemia are now discussed.

### Activation of Neuronal Glutamate Receptors

Hypoglycemia causes neuronal depolarization, resulting in a significant elevations in brain extracellular glutamate concentrations (46). It has been demonstrated that the ablation of presynaptic glutamatergic terminals prevents the rise of glutamate in the extracellular space and hypoglycemic neuronal death (47). In addition, impaired glutamate astrocyte reuptake causes an increased glutamate level leading to an accumulation of aspartate in brain tissues (48). As a consequence, increased levels of aspartate may activate some glutamate receptors subtypes, contributing to excitotoxicity. The sustained glutamate receptor activation is the first step of the process leading to neuronal cell death.

### Oxidative Stress

Oxidative DNA damage plays a critical role in neuronal cell death. Mitochondria have been implicated as a source of reactive oxygen species (ROS) in many disorders, and mitochondria taken from a hypoglycemic brain exhibit an increased capacity to generate ROS in response to glutamate excitotoxicity. However, ROS can also be generated by several other sources. NADPH oxidase is an enzyme studied in neutrophils which synthesizes superoxides. It has been observed that superoxide production in neuron cells affected by hypoglycemia occurs mainly during glucose reperfusion.

Moreover, the rate of superoxide production is influenced by blood glucose concentrations achieved in the immediate post-hypoglycemic period (49). The selective vulnerability of brain regions to hypoglycemia has been demonstrated. The II and III superficial layers of the cerebral cortex, the dentate gyrus, the subiculum, the CA1 regions in the hippocampus, and the caudate –putamen, emerged as the main susceptible areas to hypoglycemic insult (50, 51). Although the physiopathology underlying this different susceptibility still remains unclear, oxidative stress seems to play a central role (52).

### Neuronal Zinc Release

Zinc (as Zn^2+^) is a neuromodulator stored within vesicles in presynaptic terminals, and it is massively released from into the extracellular space during pathological conditions such as ischemia, seizure, brain trauma, and hypoglycemia (49, 53). Zinc significantly affects the activity of many receptors including NMDA, GABA-A, ATP, and glycine receptors as well as voltage-gated Na+ and Ca2+ channels and it has been associated with the promotion of neuronal death (53, 54). It induces profound mitochondrial dysfunction triggering mitochondrial depolarization and leading to PARP-1 activation, probably as a result of increased production of reactive oxygen species (ROS) (55, 56). In addition, it has been observed that zinc contributes to neuronal energy failure by glycolisis inhibition at GAPDH level (57).

### Activation of Poly-ADP-Ribose Polymerase–1 (PARP1)

PARP-1 has a catalytic domain that shows structural homologies to other ADP-ribosyl-transferase enzymes. It uses the ADP ribose group of NAD+ to form branched ADP ribose polymers on specific acceptor proteins near DNA strand breaks or kinks. These polymers facilitate the DNA repair process and prevent chromatid exchange. During oxidative stress, reactive species damage all cellular components, including nucleic acids. PARP-1 activation takes place in order to facilitate the DNA repair machinery. While adequate PARP activation facilitates DNA repair, extensive PARP activation, caused by extensive damage from a sustained action of glutamate, induces mitochondrial permeability transition and mitochondrial damage that culminates in cell death. Moreover, activation of PARP-1 consumes cytosolic NAD+ required for glycolytic process; therefore, capacitating neurons to utilize glucose because of PARP-1 activation, even when glucose availability is restored. Pyruvate and other non-glucose substrates can be metabolized without cytosolic NAD+ and can rescue cells from PARP-1 induced cell death (35).

## Blood Glucose Variability and Brain Damage

Cerebral blood flow (CBF) is regulated by neurons and astrocytes. Under resting conditions, local CBF is highest in brain regions with the highest local glucose metabolism. During functional activation, the increase in local CBF usually correlates with an increased cerebral metabolic rate of glucose (34). Neurotransmitter-mediated signaling, particularly by glutamate, plays a key role in regulating CBF, and much of this control is mediated by astrocytes. Traditionally, it was thought that active neurons generate a metabolic signal (hypoxemia, hypoglycemia, or hypercapnia), which triggers an increase in blood flow.

To sustain neuronal function, the brain has evolved “neurovascular coupling” mechanisms to increase the flow of blood to regions where neurons are independently active of glucose metabolism-induced signaling. However, it seems that changes in the lactate levels and its metabolic products may be at least partially responsible for vasodilatation during neuronal activation. During acute hypoglycemia, resting CBF only increases significantly when blood and brain glucose are dramatically reduced. Neither hyperglycemia nor mild/moderate hypoglycemia significantly changes the blood flow responses to functional activation. Giordani et al. demonstrated that acute hyperglycemia induced a reduction in cerebral vasomotor reactivity, both in normal control subjects and in patients with diabetes mellitus (58). To date, there are still no conclusive studies on the clinical impact of neonatal glycemic variability on cerebral flow and neurological development.

## Magnetic Resonance Imaging and Topography of The Lesions

**Figure 1:**
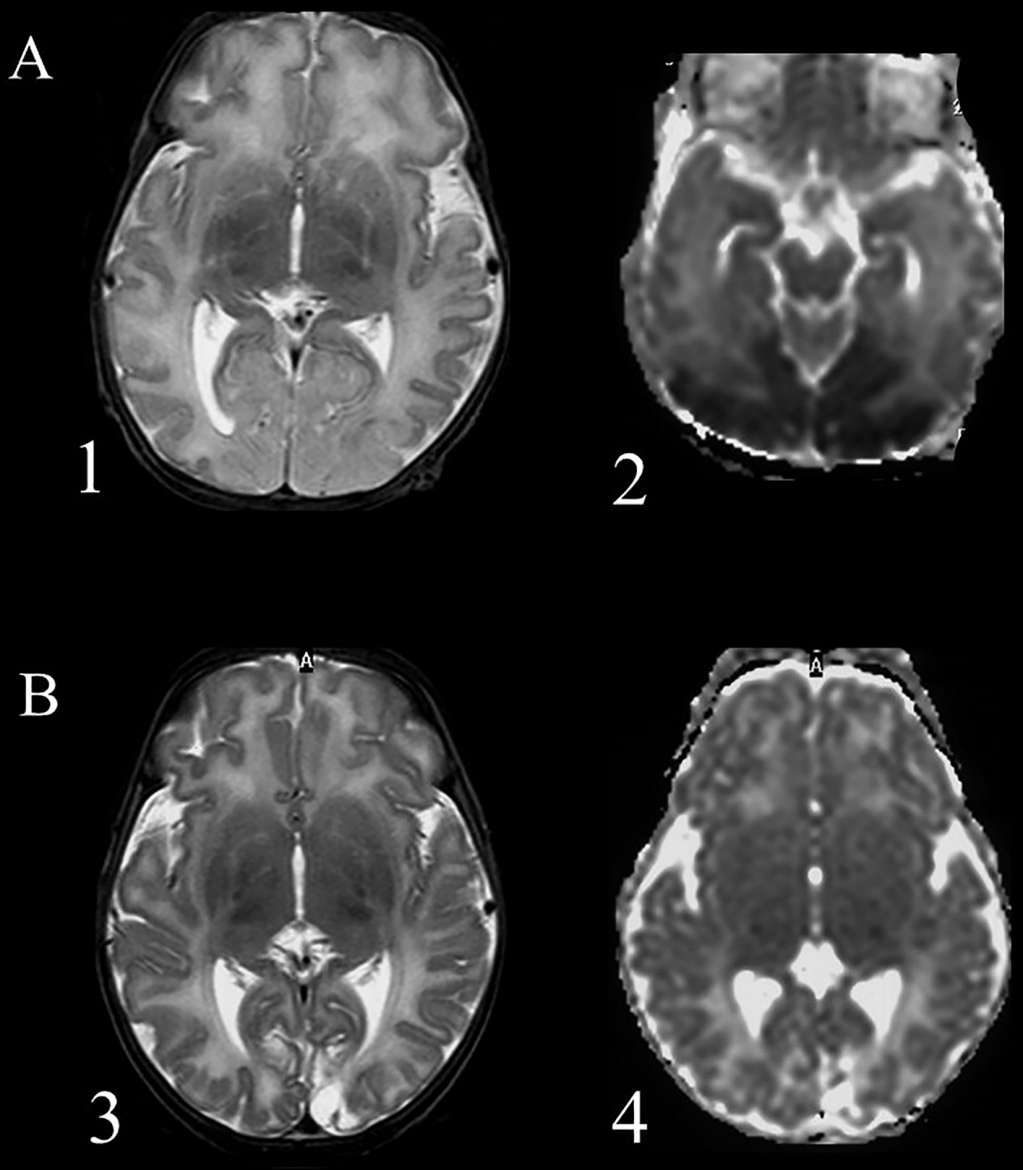
Magnetic resonance imaging of a term newborn with apnea and seizures associated with severe hypoglycemia. **(A)** scans performed after 5 days from the event (acute phase), 1 = axial T2 weighted imaging showing an extensive cortical and subcortical damage, manly in the occipital lobes, with loss of cortical differentiation; 2 = Diffusion weighted imaging shows a large posterior subcortical area of reduction of the apparent diffusion coefficient. **(B)** scans performed after 5 weeks (chronic phase): 3 = Presence of an occipital atrophic area, more extended within the left occipital lobe; 4 = Diffusion weighted imaging shows an increased water diffusion in the affected areas and a smaller cavitation.

Another controversial topic in the field of neonatal hypoglycemia is the correlation between hypoglycemic insult and brain damage as visualized by magnetic resonance imaging (MRI), and further correlation of brain damage with the outcome. Occipital lobe damage is thought to predominate, with case series describing patterns of posterior injury evident already in the acute phase on diffusion-weighted imaging (DWI), which correlated with significant structural injury later on (**Figure 1**). Moreover, the predilection for occipital and, to a minor extent, parietal zones in the hypoglycemic brain has been demonstrated in various studies by computed tomography, MRI, and single-photon emission computed tomography blood flow scans (59–64). In a study including 45 term infants with hypoglycemia, diffusion restriction in the mesial occipital poles was observed in half of the cohort with early imaging (65).

Reduction of regional cerebral glucose use, or a local expression deficit of the glucose membrane transporter proteins, have been named as possible contributors to the localization of damage (64). Another factor in play could be active synaptogenesis and axonal migration in the occipital lobe, as well as elevated levels of aspartate stimulating newly developed receptors for excitatory amino acids, causing selective death of the postsynaptic neurons (66). A MRI study in patients with symptomatic hypoglycemia associated with metabolic disease showed a correlation between pattern of damage and age at clinical presentation, suggesting a correlation with brain maturation processes: parieto-occipital white matter lesions were observed in infants with hypoglycemia occurring from the neonatal period to 6 months of age, while the basal ganglia damage and parieto-temporal cortex involvement were present in older infants (67).

On the other hand, several reports suggest that the brain damage connected with neonatal hypoglycemia could be more heterogeneous than previously thought. In a study of 35 term symptomatic infants, white matter abnormalities were not limited to the posterior regions. Basal ganglia/thalamic abnormalities, hemorrhages, and middle cerebral artery infarctions were seen, and cortical involvement was present in half of the cases. In the cohort of that study, 65% of infants presented impairments related to the white matter damage involvement at 18 months of age (68). Another study investigating the effect of hypoglycemia in a cohort of 94 term neonates at risk for neonatal encephalopathy has observed that the only type of brain lesion significantly associated with hypoglycemia was injury in the corticospinal tract (odds ratio 3.72, 95% CI 1.02 – 13.57, P=0.047) (8).

## Neurodevelopmental Outcomes in Infants With Hypoglycemia

Neonatal hypoglycemia has been associated with various forms of neurological impairment, including developmental delay, seizures, visual processing problems, and cognitive difficulties (**Table 2**), but many questions regarding which patients are most at risk, imaging-outcome correlation, or follow-up options remain unanswered (68, 69).

**Table 2:** A summary of cited studies regarding neurodevelopmental outcomes and other brain injuries in infants with neonatal hypoglycemia.

Study	Study type	Cohort year	Study population	Key imaging findings	Key outcome findings
Caraballo, 2004 (66)	retrospective cohort	1990–2003	15 infants with epilepsy and/or posterior cerebral lesions and NH	13/15 patients presented parieto-occipital lesions	1 patient: no seizures; 12 patients: focal seizures and posterior abnormalities on the EEG, the majority with a good outcome; 2 patients: encephalopathy with refractory seizures (after prolonged refractory NH).
Filan, 2006 (64)	retrospective cohort	2004	4 term and late-preterm infants with NH	Abnormalities detected in occipital and parietal cortex and white matter, corpus callosum, and optic radiations.	At 1 year of age, one case presented microcephaly, gross motor delay, and visual impairment, while other 3 cases had normal follow-up at 9 months.
Burns, 2008 (68)	retrospective cohort	1992–2006	35 term infants with symptomatic NH and 229 controls	White matter abnormalities observed in 94% of infants with NH, with predominantly posterior pattern in 29% of cases. Cortical involvement present in 51% of cases, basal ganglia/thalamic abnormalities in 40%, white matter hemorrhages in 30%, and middle cerebral artery infarctions observed in 3 infants.	At 18 months of age, 65% of infants presented impairments related to the white matter damage. Early MRI findings were more instructive than severity or duration of hypoglycemia in predicting neurodevelopmental outcomes.
Tam, 2008 (65)	retrospective cohort	2000–2005	45 neonates of different gestational ages with NH	Diffusion MRI restriction in the mesial occipital poles observed in ½ of term infants with early imaging.	No long-term visual loss in infants with single-day NH. Cortical visual loss in 1/3 of infants with NH lasting ≥2 days.
Kerstjens, 2012 (71)	community-based stratified cohort	2002–2003	832 moderately preterm infants	–	Around 4 years of age, NH was found to increase the risk of developmental delay in moderately preterm-born children.
Tam, 2012 (8)	prospective cohort	1994–2010	94 term neonates at risk for neonatal encephalopathy	NH associated with a 3.72-fold increased odds of corticospinal tract injury (P=0.047).	At 1 year of age, NH was assosiated with 4.82-fold increased odds of one-point worsened neuromotor score (P=0.038) and a 15-point lower cognitive and language score on the Bayley Scales of Infant Development (P=0.015).
Wong, 2013 (83)	cohort	2004–2010	179 term infants with neonatal encephalopathy	Specific imaging features for both NH and HIE can be identified. Selective edema in the posterior white matter, pulvinar, and anterior medial thalamic nuclei were most predictive for NH, while no injury (36%) or a watershed (32%) pattern of injury were seen more often in severe NH.	–
van der Aa, 2013 (85)	retrospective	2000–2012	18 infants with perinatal arterial ischemic stroke in the PCA territory	7/18 patients diagnosed with NH → possible relation between hypoglycemic brain damage and posterior stroke.	–
Gataullina, 2013 (67)	retrospective cohort	–	50 patients with symptomatic metabolic hypoglycaemia at 1 day–5 years	Parieto-occipital white matter lesions were observed in infants with hypoglycaemia occurring from the neonatal period to 6 months of age.	Clinical sequelae were severe (global psychomotor delay, microcephaly, motor deficit, lack of visual contact, and/ or pharmacoresistant epilepsy) in 22 children and mild (speech delay, learning difficulties, and pharmacosensitive epilepsy) in 13 others; 15 children experienced no sequelae.
Fong, 2014 (7)	retrospective	1996–2012	11 patients with seizures beyond infancy after NH	All children presented gliosis with or without cortical atrophy in the occipital lobe with or without parietal lobe involvement.	Despite having bilateral occipital brain injury and neurological disability, 6/11 children with epilepsy after NH had infrequent and potentially age-limited focal seizures.
McKinlay, 2015 (69)	prospective cohort	2006–2010	404 term and late preterm neonates at risk for NH treated to maintain blood glucose ≥47 mg/dl	–	At 2 years of age, no increased risk of neurosensory impairment or processing difficulty in infants with NH. No correlation between the lowest blood glucose concentration, number of hypoglycemic episodes, or episodes of unrecognized hypoglycemia, and the given outcome.
Kaiser, 2015 (74)	retrospective population-based cohort	1998	1943 infants of different gestational ages	–	At 10 years of age, an association was found between early transient hypoglycemia and decreased probability of proficiency on literacy and mathematics achievement tests.
Goode, 2016 (72)	secondary analysis of a longitudinal study	1985	743 preterm infants stratified into 4 groups by glucose level	–	No significant differences in cognitive or academic skills at 3, 8, and 18 years of age were observed in preterm-borninfants stratified by glucose level.
Basu, 2016 (82)	secondary analysis of a randomized study	1999–2002	214 neonates with HIE	–	A greater risk of unfavorable outcome observed in infants with HIE and NH
McKinlay, 2017 (75)	prospective cohort	2006–2010	477 term and late preterm neonates at risk for NH treated to maintain blood glucose ≥47 mg/dl	–	At 4.5 years of age, NH was not assosiated with increased risk of combined neurosensory impairment, but was assosiated with increased risk of poor executive and visual motor functions. Highest risk in children exposed to severe, recurrent, or clinically undetected NH.
Basu, 2018 (84)	secondary analysis of a prospective study	2008–2016	178 neonates with HIE	An association observed between early NH in infants with HIE and watershed or focal-multifocal injury on MRI.	–

NH, neonatal hypoglycemia; HIE, hypoxic-ischemic encephalopathy; PCA, posterior cerebral artery.

A recent meta-analysis considering neurodevelopmental outcomes after neonatal hypoglycemia underlined the need for additional studies to determine the best strategies for improving long-term outcomes in neonates at risk: the authors found a correlation between hypoglycemic events and an increased risk of visual motor impairment and executive dysfunction in early childhood and an increased risk of literacy and numeracy problems in later childhood, but the quality of evidence was low, and data on long-term outcomes were not available (70).

One of the reasons for disagreeing evidence in the field could be the difficulty associated with defining neonatal hypoglycemia, starting from debates on the thresholds for intervention, including a need to take into consideration person-to-person variability in glucose homeostasis and reaction to hypoglycemia (68). For example, in a large cohort study involving 832 moderately preterm children, different clinical parameters were evaluated in association with the developmental delay at 4 years of age, and only NH was found to increase the risk of developmental delay (71). Another cohort study that included 745 preterm infants, had patients stratified into 4 groups by glucose level, and cognitive, academic, and behavioral outcomes were assessed at 3, 8, and 18 years of age. In this study, the authors did not observe any significant differences in cognitive or academic skills between the groups at any age (72). In term infants, a small study evaluated neurodevelopmental outcomes at 18 months of age in 35 patients with symptomatic neonatal hypoglycemia. No relationship was found between the severity or duration of hypoglycemia and outcomes (68).

Moreover, while many authors agree that severe, persistent hypoglycemia can cause seizures and brain injury, the prognostic meaning of transient hypoglycemia remains controversial (73, 74).

A big retrospective population-based cohort study involving 1943 infants of different gestational ages found a clear association between early transient hypoglycemia and decreased probability of proficiency on literacy and mathematics achievement tests at age 10 years, leading to considerable conversation regarding the possible usefulness of universal newborn glucose screening (74). At the same time, one of the biggest prospective cohort studies on the topic (CHYLD Study), which included 404 late preterm and term neonates at risk for hypoglycemia, studied infants who were treated to maintain a blood glucose concentration of at least 47 mg/dl. Neurodevelopmental follow-up was assessed first at 2 years, and then at 4.5 years of age (69, 75). At two years, those with treated hypoglycemia were observed to not exhibit an increased risk of neurosensory impairment or processing difficulty. No correlation was found between the lowest blood glucose concentration, number of hypoglycemic episodes, or episodes of unrecognized hypoglycemia, and the given outcome (69). At 4 and a half years, hypoglycemia was not associated with an increased risk of combined neurosensory impairment, but was associated with an increased risk of low executive function and visual motor function, with the highest risk in children exposed to severe, recurrent, or clinically undetected hypoglycemia.

Another interesting result of that study was the association of cognitive delay with higher glucose concentration and less glucose stability after treatment, observed as a longer time outside the range of 54-72 mg/dl in the first two days after birth. Authors suggest that glucose reperfusion injury in the case of overly rapid or overly intense correction of hypoglycemia may exacerbate oxidative stress (76), even if data on biological effect of glycemic variability in neonates is almost absent in literature (77).

## Visual Deficits

Visual outcomes connected with neonatal hypoglycemia seem to depend on the severity and length of hypoglycemic insult, even if various comorbidities, difficulties in testing vision, and frequent absence of school-age follow-up makes the interpretation of the present data difficult (78). MRI that was performed promptly after insult could be of help in predicting visual outcomes. In a study involving 25 neonates of different gestational ages, diffusion restriction in the mesial occipital poles was associated with cortical visual deficits. It is interesting to note that restricted diffusion was observed in term but not in preterm infants. In the same study, visual outcomes seemed to correlate with the duration of the hypoglycemic insult: cortical visual loss was documented for one-third of infants with hypoglycemia measured over 2 days, while no long-term visual loss was observed in infants with neonatal hypoglycemia documented on a single day only (65).

## Epilepsy

Epilepsy with seizures protracting beyond infancy is one of most threatening consequences of NH, with the majority of cases described as being of occipital origin (79, 80). The presence of epilepsy is rarely the only symptom of previous neonatal hypoglycemia, with patients often presenting with other neurological problems, such as visual disturbances, developmental delay, or pervasive developmental disorders. As for other outcomes, electroclinical features and an evolution of the disease seem to depend on the severity of the initial insult and the eventual presence of other comorbidities (66). Even if cases of epileptic encephalopathy with refractory seizures has been described, the literature suggests that in a lot of cases, epilepsy can be mild, focal, and age-limited with favorable outcomes in adolescence (7).

## Hypoglycemia and Hypoxic-Ischemic Encephalopathy

Negative influence of hypoglycemic insult on future neurodevelopment is exacerbated by the presence of other comorbidities most significantly by hypoxic-ischemic encephalopathy (HIE). Hypoglycemia tend to have both an additive and potentiating role in producing brain injury. The number of ATP molecules that can be produced from one molecule of glucose goes down drastically with the transition from aerobic to anaerobic conditions. Thus, hypoxemia and ischemia increase cerebral demand for glucose, exposing brain cells to a significantly increased risk of damage in the case of low blood glucose levels (66). An interesting study by Basu et al. compared 60 infants with HIE to 60 controls, exploring the relationship between plasma glucose level and the neurological status of the patients. Authors have observed lower mean plasma glucose level in the asphyxiated group when compared to the controls. Furthermore, a negative linear correlation between the glucose level and different stages of HIE was observed, which led to the conclusion that the severity of encephalopathy varies with the severity of hypoglycemia (81). Moreover, the CoolCap Study of therapeutic hypothermia for neonates with HIE observed a greater risk of negative outcome in infants who presented with hypoglycemia (82).

Even if HIE and hypoglycemia can have a combined negative effect on the brain, it seems possible to diversify MRI patterns of the two types of injury. Indeed, in a study including 179 term infants, authors were able to predict hypoglycemia from only the MRI result (selective edema in the posterior white matter, pulvinar, and anterior medial thalamic nuclei) with a positive predictive value of 82% and negative predictive value of 78% (83). Another study including 178 neonates with HIE showed a distinct association between early glycemic profile in infants with HIE and specific patterns of brain injury on MRI, with predominant watershed or focal-multifocal injury being more frequent in infants with hypoglycemia (84).

## Hypoglycemia as a Risk Factor for Other Brain Lesions

Another question that needs to be addressed is a possible role of alterations in glucose metabolism as a risk factor of other brain lesions. An interesting result comes from work by van der Aa et al., which described a cohort of infants with perinatal arterial ischemic stroke in the territory of the posterior cerebral artery. Seven patients out of 18 included in the study were diagnosed with neonatal hypoglycemia, leading to doubts about the possible relation between hypoglycemic brain damage and posterior stroke. A prevalent pattern of injury observed in the study was a unilateral occipital damage with a sharp demarcation line, different from what is habitually described for the hypoglycemic brain. The authors suggested that hypoglycemia may have acted as a risk factor for stroke development, underlining that the exact role of hypoglycemia in the pathogenesis of posterior stroke requires further investigation (85).

In preterm infants, the risk of germinal matrix hemorrhage - intraventricular hemorrhage is reported to be increased in case of hyperglycemia and higher glycemic variability, while no association seems to be present with hypoglycemia (86, 87). On the other hand, when intraventricular hemorrhage occurs atypically in term infants, hypoglycemia could be an underlying risk factor, as in 3 out of 35 term symptomatic infants with in one MRI study (68).

It has been suggested that less mature white matter could be more vulnerable to the negative effect of hypoglycemia, although no direct evidence of this possibility seem to exist at the moment. It is interesting to note how multiple punctate white matter lesions compatible with hemorrhage in the periventricular white matter were described both in term infants suffering from hypoglycemia and in extremely preterm infants (68, 88). Nevertheless, a role of hypoglycemia as a risk factor for punctate white matter lesions in preterm infants has not been yet investigated.

## Prevention of Neonatal Brain Damage Hypoglycemia

Although the actual risk of neurologic injuries and abnormal outcomes, prevention of neonatal hypoglycemia still remains challenging. Neonatal hypoglycemia is frequently asymptomatic or associated with aspecific symptoms, and a precise glycemic threshold for brain injury development is not clearly established. The screening programs available to date displays substantial issues, including overtreatment of the physiologic glycemic reduction after birth and interference with breastfeeding, excessive blood sampling in at-risk euglycemic infants and missed early diagnosis of hypoglycemia in infants not included in the at-risk categories (89). Screening strategies in asymptomatic infants are suggested in the presence of several maternal or neonatal conditions possibly affecting the metabolic adaptation after birth (**Table 3**) (14, 90–92).

**Table 3:** Risk factors associated with hypoglycemia.

Maternal conditions
Diabetes or altered glucose tolerance Preeclampsia/eclampsia or hypertension Earlier pregnancy with a large infant Treatment with tricyclic antidepressants Treatment with serotonin-norepinephrine reuptake inhibitors Treatment with tocolytics Treatment with beta blockers Drug abuse Family history of a genetic form of hypoglycemia Congenital syndromes (eg, Beckwith-Wiedemann)
**Infant conditions**
Premature birth (<37 weeks) Small for gestational age (SGA < 10th percentile) Intrauterine growth restriction (IUGR) Large for gestational age (LGA >90th percentile) Discordant twin (weight discordance > 10%) Low birth weight (< 2,500 g) Sepsis Perinatal hypoxia-ischemia Rhesus disease Polycythemia Hypothermia (temperature ≤ 35°C) Respiratory distress Erythroblastosis fetalis Beckwith-Weidman Syndrome Delayed start of breastfeeding

Routine measurements of blood glucose concentrations are recommended for infants with a risk factor for compromised metabolic adaptation or infants showing signs and symptoms suggestive of hypoglycemia. Many guidelines considered universal glucose monitoring for hypoglycemia in healthy, asymptomatic, term infants born after uncomplicated pregnancy and delivery to be an inappropriate procedure. Therefore, glucose screening should be reserved only for infants at risk. Glucose monitoring should be initiated within 3 h of life, or at any time infants showed symptoms of hypoglycemia. The decision to discontinue blood glucose monitoring depends on the glycemic trend and the treatment needed (2).

Early and exclusive breastfeeding meets the nutritional and metabolic needs of healthy, term newborn infants. A prompt initiation of breastfeeding immediately after birth reduces the risk of hypoglycemia. In addition, skin to skin practice help maintaining an adequate body temperature thus reducing energy expenditure and glucose consumption, while stimulating milk production (93, 94). Infants at risk for hypoglycemia must be fed within the first hour of life, before performing the first plasma glucose detection, and supplementary feeding with mother’s own breast milk or infant formula is recommended for all infants at risk for hypoglycemia. On the contrary, feeding with dextrose solution in the postnatal period may cause negative metabolic effects including increased insulin secretion, decreased glucagon secretion, delay of gluconeogenesis and ketogenic homeostasis. Prevention with dextrose gel has been recently proposed as an alternative to early supplementary feedings, but its efficacy on neonatal hypoglycemia prevention is still unclear (95).

It is commonly accepted that symptomatic infants should be promptly treated, in order to avoid severe and prolonged hypoglycemia which may result in neurologic injury (68). On the other hand, the management of hypoglycemia in asymptomatic infant poses more problems.

Frequent milk feedings associated with repeated glucose measurements represent the current standard treatment for asymptomatic hypoglycemia in high risk neonates (96). In infants with very low blood glucose or persistent hypoglycemia in spite of feedings, it is commonly accepted to start intravenous glucose administration. However, as improvement in long term outcomes following a rapid correction of asymptomatic hypoglycemia still remains unclear, and may expose to the risk of brain damage associated with a sudden increase in blood glucose levels and high glucose variability, we stress the importance of oral correction over the intravenous route in asymptomatic hypoglycemia.

Several screening and treatment programs have been proposed, with slight differences on operative thresholds and treatment strategies, aiming at the prevention of neonatal hypoglycemia through the following interventions:

- early feeding and blood glucose monitoring in infants considered at risk;
- use feeding supplementation as the first step to treat mild and asymptomatic hypoglycemia;
- promptly start intravenous dextrose infusion in case of severe, prolonged, and symptomatic hypoglycemia.

Though this approach seems the most reasonable, we also suggest to:

- Carefully evaluate maternal and pregnancy history and perinatal comorbidities for a prompt identification of at-risk infants;
- Perform a complete physical evaluation to exclude even mild symptoms of hypoglycemia;
- Carefully calculate the dextrose infusion rate to avoid dangerous glycemic oscillations;
- Intensively support breastfeeding through frequent and personalized breastfeeding interventions;
- Closely monitor glucose levels to titrate dextrose dose in order to avoid hyperglicemia, significant glycemic oscillations or recurrent episodes of hypoglycemia, which are associated with an increased risk of neurologic damage (71).

## Discussion

Despite its significant prevalence, neonatal hypoglycemia remains a challenging condition. The transition of glucose homeostasis at birth leads to a temporary reduction of blood glucose levels in the first hours of life which is rarely symptomatic in healthy infants. However, the absence of clear blood glucose cut-off values in the first hours of life and the lack of universally accepted guidelines render its management largely uncertain. At the same time, though a causative role of hypoglycemia in brain damage has been demonstrated, a precise damage topography and the relationship between the entity and length of hypoglycemia and the extent of brain injury have not been clarified yet. Interpersonal variability in glucose homeostasis and the confounding effects of comorbidities especially in preterm infants may largely explain the uncertain results of the studies. In addition, the effect of glycemic oscillations often associated with hypoglycemia treatment could play an important role in increasing the risk of brain damage, possibly through enhancing oxidative stress responses.

Although few evidences are available to precisely establish operative guidelines to reduce hypoglycemia-related brain damage, we suggest to carefully assess the possible risk factors for hypoglycemia, to intensively support breastfeeding and to precisely titrate the dextrose dose.

Collaborative studies involving specific neonatal subpopulations together with comparable animal models may help discovery new insights in brain damage after neonatal hypoglycemia.

## Author Contributions

LR, LD, and GB contributed conception of the study. GP, GB, AP, CT, PM, and MaM performed a literature search and wrote sections of the manuscript. LD, MoM, and DM critically revised the manuscript and wrote section of the manuscript. AR provided the brain magnetic resonance imaging and wrote a section of the manuscript. All authors contributed to the article and approved the submitted version.

## Conflict of Interest

The authors declare that the research was conducted in the absence of any commercial or financial relationships that could be construed as a potential conflict of interest.

## Abbreviations

ADP, adenosine-di-phosphate; ATP, adenosine-tri-phosphate; BBB, blood-brain-barrier; CBF, cerebral blood flow; DWI, diffusion-weighted imaging; GABA, gamma-aminobutyric acid; GE, glucose-excited; GI, glucose-inhibited; GLUT-1, glucose transporter-1; GLUT-3, glucose transporter-3; HIE, hypoxic-ischemic encephalopathy; LGA, large-for-gestational age; MRI, magnetic resonance imaging; NADPH, nicotinamide adenine dinucleotide phosphate; NAD+, nicotinamide adenine dinucleotide; NH, neonatal hypoglycemia; NMDA, *N*-methyl-d-aspartate; PARP-1, poly-ADP-ribose polymerase-1; ROS, reactive oxygen species; SGA, small-for-gestational age.
